# Correction: Psychological distress related to the emerging COVID-19 pandemic and coping strategies among general population in Egypt

**DOI:** 10.1186/s42506-022-00126-0

**Published:** 2022-12-12

**Authors:** Manal Mohamed Elkayal, Mahmoud Abdel Hameed Shahin, Rasha Mohammed Hussien

**Affiliations:** 1 Department of Psychiatric and Mental Health Nursing, Faculty of Nursing, Zagazig University, Zagazig, Egypt; 2 Department of Critical Care Nursing, Mohammed Al-Mana College for Medical Sciences, Dammam, Saudi Arabia; 3 Department of Psychiatric and Mental Health and Community Health Nursing, College of Nursing, Qasim University, Buraydah, Saudi Arabia

**Correction: J Egypt Public Health Assoc 97, 3 (2022)**

**https://doi.org/10.1186/s42506-021-00100-2**

Following publication of the original article [1], we have been notified that affiliation 3 of author Rasha Mohammed Hussein was mentioned incorrectly. Correct affiliation should be as per below:

Department of Psychiatric and Mental Health and Community Health Nursing, College of Nursing, Qasim University, Buraydah, Saudi Arabia.
